# National chlorhexidine coverage and factors associated with newborn umbilical cord care in Bangladesh and Nepal: a cross-sectional analysis using household data

**DOI:** 10.1186/s40748-024-00182-8

**Published:** 2024-06-07

**Authors:** Kavita Singh, Elizabeth Simmons, Bliss Garriga, Grace Hoover, Rashida E. Ijdi, Ashish KC

**Affiliations:** 1 Data for Impact (D4I), University of North Carolina at Chapel Hill, Chapel Hill, NC USA; 2 Department of Maternal and Child Health, University of North Carolina Gillings School of Global Public Health, Chapel Hill, NC USA; 3 Carolina Population Center, University of North Carolina at Chapel Hill, Chapel Hill, USA; 4 School of Public Health and Community Medicine, University of Gothenburg, Gothenburg, Sweden

**Keywords:** Chlorhexidine, Neonatal mortality, Bangladesh, Nepal

## Abstract

**Background:**

Preventable newborn deaths are a global tragedy with many of these deaths concentrated in the first week and day of life. A simple low-cost intervention, chlorhexidine cleansing of the umbilical cord, can prevent deaths from omphalitis, an infection of the umbilical cord. Bangladesh and Nepal have national policies promoting chlorhexidine use, as well as routinely collected household survey data, which allows for an assessment of coverage and predictors of the intervention.

**Methods:**

We used data from the 2017–2018 Bangladesh Demographic and Health Survey and the 2016 Nepal Demographic and Health Survey, two large-scale nationally representative household surveys. We studied coverage of single application of chlorhexidine to the umbilical cord of newborns born in the past year using descriptive, bivariate and multivariable analyses. Key predictors of newborns receiving chlorhexidine cleansing, including socio-economic factors, healthcare related factors and the application of harmful and nonharmful substances, were explored in this study.

**Results:**

Coverage of chlorhexidine cleansing was 15.0% in Bangladesh and 50.7% in Nepal, while the application of a harmful substance was 16.9% in Bangladesh and 22.6% in Nepal. Results from the multivariable analyses indicated that delivery in a health facility was strongly associated with a newborn’s receipt of chlorhexidine in both countries (Bangladesh: OR = 2.23, *p* = 0.002; Nepal: OR = 5.01, *p* = 0.000). In Bangladesh, delivery by Cesarean section and application of another non-harmful substance were significantly and positively associated with the receipt of chlorhexidine. In Nepal antenatal care was significantly and positively associated with chlorhexidine, while application of a harmful substance was significantly and negatively associated with receipt of chlorhexidine. Maternal education, urban/rural residence, religion and sex were not significant in the multivariable analysis. Wealth was not a significant factor in Bangladesh, but in Nepal newborns in the two highest wealth quintiles were significantly less likely to receive chlorhexidine than newborns in the lowest wealth quintile.

**Conclusion:**

As Bangladesh and Nepal continue to scale-up chlorhexidine for newborn umbilical cord care, additional focus on newborns born in non-facility environments may be warranted. Chlorhexidine cleansing may have the potential to be an equitable intervention, as newborns from the poorest wealth quintiles and whose mothers had less education were not disadvantaged in receiving the intervention in these two settings.

## Background

A tragic 2.4 million newborns died in the year 2022 [1] and many of these deaths could have been prevented with known interventions. Most neonatal deaths (75%) occur within the first week of life, and a large proportion of these deaths occur on the very first day of life [2]. Neonatal mortality accounts for 47% of under-five mortality [1] with the primary causes of death being preterm birth, intrapartum-related complications, infections and congenital issues [2]. `The mortality rate from omphalitis, an infection of the umbilical cord, has been estimated to be between 7% and 15% [3]. *Staphylococcus aureus, Streptococcus pyogenes, Escherichia coli, Klebsiella pneumoniae*, and *Proteus mirabilis* cause most cases of omphalitis and often originate from the skin or the gut [3–5]. Thus, hygienic umbilical cord care is essential in preventing infections and deaths from omphalitis.

Chlorhexidine digluconate is a low-cost antiseptic that is found in products such as hand sanitizers and mouthwash at low doses. At a higher 7.1% dose, it can be used for newborn umbilical cord care to avert omphalitis and neonatal deaths. Clinical trials conducted in South Asia [6–8] revealed that 7.1% chlorhexidine application was associated with a 23% reduction in neonatal mortality [9]. Based on these findings, in 2014 the World Health Organization (WHO) recommended daily application of chlorohexidine during the first week of life (including immediately after cutting the umbilical cord) for newborns born at home in settings with high neonatal mortality (defined as over 30 deaths/1000 live births). For newborns born in health facilities or in lower mortality settings, clean and dry cord care was recommended. In these settings chlorohexidine was only recommended in situations where harmful substances might otherwise be applied [10]. In the 2022 WHO recommendations, application of chlorohexidine was recommended only in settings where potentially harmful substances are traditionally applied to the umbilical cord. Otherwise, clean and dry cord care was promoted as the gold standard [11]. This change was made based on later studies that did not find that chlorohexidine resulted in significantly lower neonatal mortality over clean and dry cord care [12, 13].

Many cultures have traditions where a substance is applied to the umbilical cord often due to beliefs that the substance could facilitate healing, cord separation or help protect the newborn from cold and illnesses [14]. In such settings, chlorhexidine can be promoted as a replacement for a harmful substance that would have otherwise been applied to the cord. Substances commonly applied to the cord include herbs, ash, charcoal, dung, clarified butter (or *ghee*) and various oils [14].

While several clinical trials have examined the effectiveness of chlorhexidine in terms of preventing newborn infections and deaths [6–8, 12, 13], this study is focused on understanding coverage of the intervention in two countries, Bangladesh and Nepal. Both countries have population-level data on chlorhexidine for umbilical cord care, as well as long-standing policies promoting the intervention with a target of 95% coverage by 2035 [15, 16]. Nepal was the first country to promote a scale-up of chlorhexidine for newborn care beginning in 2011 with a focus on 49 of 75 districts. The second phase began in 2014 and was intended to be a scale-up to the remaining districts [17, 18]. Bangladesh began promoting universal chlorhexidine for umbilical cord care in 2014, though the initial focus was actually on health facilities [19]. It should be noted that both countries promote a single application of chlorhexidine immediately after birth and not for a full week as per the WHO guidelines [20, 21]. The objectives of this paper are to describe the coverage of chlorhexidine for newborn cord care in the two countries and to analyze factors associated with receipt of the intervention in order to better inform programs and interventions.

## Methods

### Setting

Bangladesh is a densely population country with a population of 173 million [22] and an area of 147,630km^2^ [23] Nepal has a population of 30.9 million [22] and an area of 147,630km^2^ [23]. Neonatal mortality in Bangladesh has fallen from 44.1 neonatal deaths per live births in 2000 to 16.0 neonatal deaths per live births in 2021 [1]. In Nepal neonatal mortality has decreased from 39 neonatal deaths per live births in 2000 to 16 neonatal deaths per live births in 2021 [1]. Skilled delivery coverage was 53.9% in Bangladesh and 64.7% in Nepal, according to 2017–2018 Demographic and Health Survey (DHS) data fror Bangladesh and the 2016 DHS for Nepal [24, 25].

### Data source

Data from the 2017–2018 Bangladesh DHS [24] and the 2016 Nepal DHS [25] were used for this study. Both of these sources of data were collected prior to the Covid-19 pandemic and were the most recent DHS data available at the time of the study. Every DHS is a nationally representative household level survey, typically conducted every five years in select countries, allowing for monitoring and evaluation of socio-economic and health factors over time. The data are representative of both urban and rural areas, and administrative subdivisions. The surveys are conducted using a two-stage cluster design, first creating Enumeration Areas (EA) from census data, and then drawing a sample of households from each EA. Within households, women 15–59 are eligible to participate in the survey [24, 25]. Some surveys conducted by the DHS also include men, and consent is obtained from respondents before the questionnaires are administered. The 2017–2018 Bangladesh DHS included 20,127 ever married women ages 15–49, while the 2016 Nepal DHS included 12,862 women ages 15–49. For both countries we restricted our sample to women of reproductive age 15–49 and their most recent live birth in the year prior to DHS data collection. We restricted to births in the past year in order to allow for enough time for the policies in each country to have been implemented. The total sample size for our study was 1690 mother-infant pairs for Bangladesh, and 961 mother-infant pairs for Nepal.

### Outcome variable

The outcome variable was application of chlorhexidine to the stump of the umbilical cord. The survey questions to obtain this outcome variable were asked differently to female respondents in each country’s DHS. In Bangladesh respondents were asked if they put anything on the umbilical cord. If they answered “yes”, there were two follow-up questions. The first follow-up questions was.

*“Did you or anyone else put chlorhexidine on the cord stump?”.* The second follow-up question was *“Other than chlorhexidine, what was applied to the cord after it was cut and dried?”* In Nepal, if a positive answer was given to whether anything was applied to the cord, then the surveyor asked what was applied, with chlorhexidine being a possible answer. A succeeding follow-up question then asked specifically about chlorhexidine application: *Was navi malam (chlorhexidine) applied to the stump at any time?* We operationalized the outcome variable as whether or not chlorhexidine was applied to the cord at any time [yes/no].

### Independent variables

We included socio-economic, biological and healthcare factors as our independent variable that might impact receipt of chlorhexidine cleansing based on the current literature. These variables are often key determinants of maternal and child health outcomes, The biological variables included maternal age (< 19, 20–34 and 35+), parity (1, 2–3 and 4+) and sex of the child (female vs. male). The socio-economic variables included maternal education (none, primary and secondary and higher) the wealth index (poorest, poorer, middle, richer, richest), type of residence (urban/rural) and subnational level units (Division for Bangladesh and Provinces for Nepal). The religion variable was unique to each country. The categories were Muslim, Hindu/other for Bangladesh and Hindu, Buddhist, and other for Nepal. All of these independent variables were self-reported by respondents in the sample with the exception of the wealth index. The wealth index is based on the owernship of assets and household structure and facilities, and these questions may have been asked to respondents in our sample or may have been asked to other members in their household.

A number of healthcare-related variables were studied including antenatal care (ANC) visits (< 4, 4+), health facility delivery (yes vs. no), and having a Cesarean section (yes vs. no). To understand the application of substances other than chlorhexidine, we explored three variables. These were another substance applied to the cord, having a non-harmful substance other than chlorhexidine applied to the umbilical cord stump and having a harmful substance applied to the umbilical cord stump. A non-harmful substance was defined as antiseptics/rubbing alcohol/methylated spirits, gentian violet or antibiotics. Other substances such as dung, ash, charcoal, and oils were classified as harmful. All the healthcare-related questions were self-reported by respondents in our sample.

### Analysis

Bivariate and multivariate analyses were performed separately for each country. The number and frequency of mother-infant dyads who did and did not apply chlorhexidine to the cord stump were compared for each independent variables, using chi-square tests. Multivariable logistic regression was used to calculate the odds ratio (OR) and 95% confidence interval (CI) for receipt of chlorhexidine cleansing for each independent variable. Other substances applied was not included in the regression models because it was highly correlated with the other non-harmful and harmful substances applied variables. The other non-harmful substance variable was not included in the regression model for Nepal due to small cell sizes. All analyses were conducted using Stata 17.

## Results

**Fig. 1:**
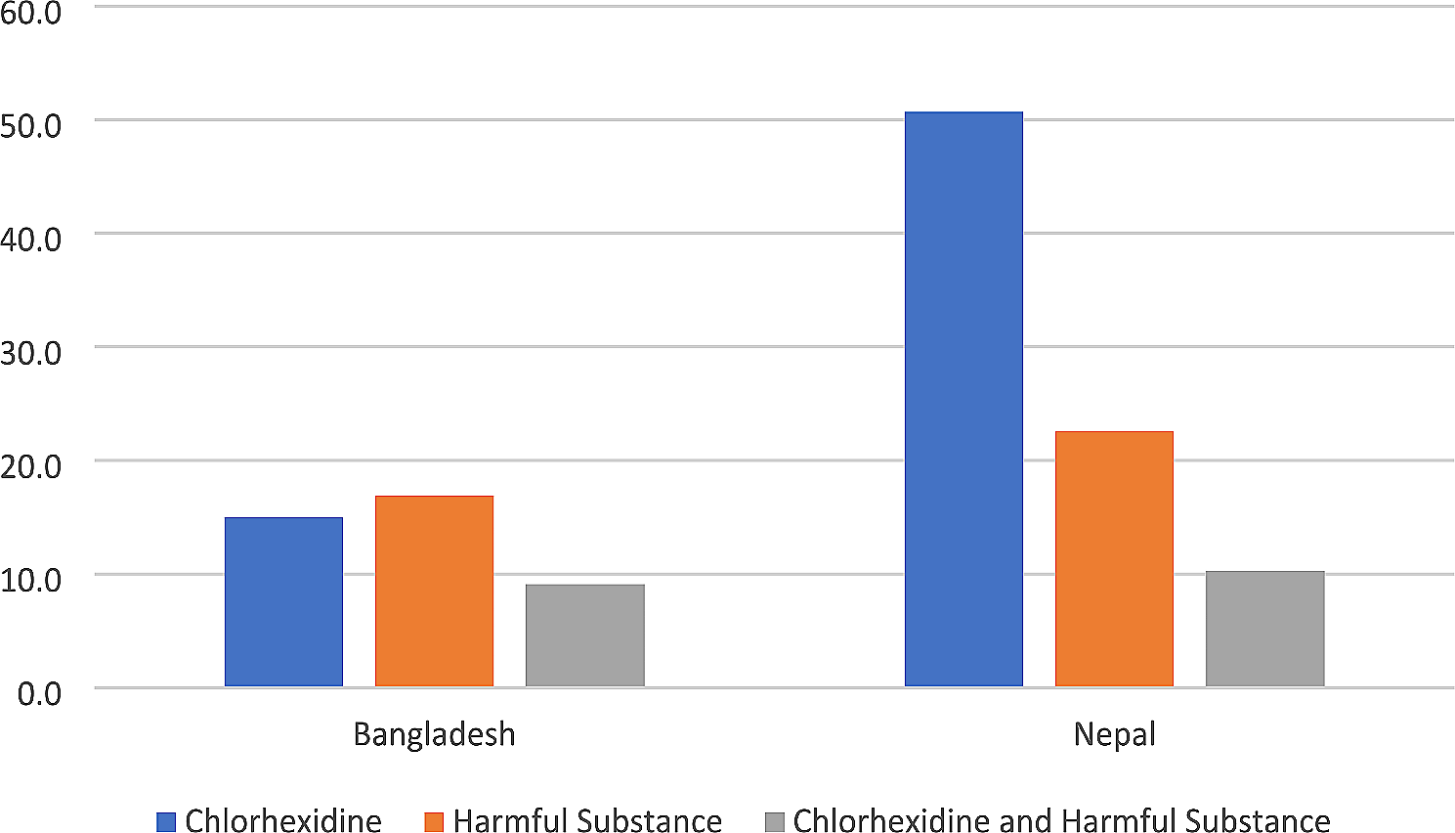
Percent of Newborns Receiving Chlorhexidine, a Harmful Substance and Both Chlorhexidine and a Harmful Substances

Figure 1 presents the percentages of newborns born in the past year who had chlorhexidine, a harmful substance or both chlorhexidine and a harmful substance to their umbilical. The percentages of newborns having chlorhexidine applied was 15.0% in Bangladesh and 50.7% in Nepal. Application of a harmful substance was 16.9% in Bangladesh and 22.6% for Nepal. In Bangladesh 9.1% of newborns received both chlorhexidine and a harmful substance, while 10.3% of newborns in Nepal received both types of substances.

Table 1 contains data for the bivariate analyses. Most women were in the 20–34 years old for both Bangladesh (68.9%) and Nepal (73.9%). There was a fairly even split for sex of the newborn in both countries, and most women (90.1% in Bangladesh and 87.3% in Nepal) were of parity one or two to three. Rural residence was more common in Bangladesh (66.9%) while urban residence was slightly more common in Nepal (55.5%). In Bangladesh 46% of respondents had a facility delivery, and in Nepal 63% of respondents had a facility delivery.

**Table 1:** Key characteristics of mothers and newborns in Bangladesh and Nepal, by receipt of chlorhexidine status

Factors	Bangladesh *n* = 1690 Was chlorohexidine applied to the cord stump?				Nepal *n* = 961 Was chlorohexidine applied to the cord stump?			
	Yes *n* = 253 *n* (col %)	No *n* = 1437 *n* (col %)	Total *n* = 1690 *n* (col %)	*p*-value	Yes *n* = 487 *n* (col %)	No *n* = 474 *n* (col %)	Total *n* = 961 *n* (col %)	*p*-value
**Maternal Age at Birth (in years)**								
13–19	67 (26.5)	383 (26.6)	450 (26.6)	0.867	108 (22.2)	112 (23.6)	220 (22.9)	0.086
20–34	173 (68.4)	991 (69.0)	1164 (68.9)		369 (75.8)	341 (71.9)	710 (73.9)	
35+	13 (5.1)	63 (4.4)	76 (4.5)		10 (2.1)	21 (4.4)	31 (3.2)	
**Parity**								
1	104 (41.3)	529 (37.1)	633 (37.8)	0.056	241 (49.9)	155 (33.3)	396 (41.8)	0.000
2–3	133 (52.8)	743 (52.2)	876 (52.3)		206 (42.6)	225 (48.4)	431 (45.5)	
4+	15 (5.9)	152 (10.7)	167 (9.9)		36 (7.5)	85 (18.3)	121 (12.8)	
**Gender**								
Male	134 (53.0)	744 (51.8)	878 (52.0)	0.727	264 (54.2)	258 (54.4)	522 (54.3)	0.945
Female	119 (47.0)	693 (48.2)	812 (48.0)		223 (45.8)	216 (45.6)	439 (45.7)	
**Mother’s Highest Level of Education**								
None	11 (4.3)	102 (7.1)	113 (6.7)	0.000	91 (18.7)	166 (35.0)	257 (26.7)	0.000
Primary	44 (17.4)	444 (30.9)	488 (28.9)		87 (17.9)	99 (20.9)	186 (19.3)	
Secondary and Higher	198 (78.3)	891 (62.0)	1089 (64.4)		309 (63.4)	209 (44.1)	518 (53.0)	
**Residence**								
Urban	95 (37.6)	465 (32.4)	560 (33.1)	0.106	299 (61.4)	234 (49.4)	533 (55.5)	0.000
Rural	158 (62.4)	972 (67.6)	1130 (66.9)		188 (38.6)	240 (50.6)	428 (44.5)	
**Religion**								
Hinduism					434 (89.1)	386 (81.4)	820 (85.3)	0.001
Islam/Buddhism	225 (88.9)	1330 (92.6)	1555 (92.0)	0.050	19 (3.9)	18 (3.8)	37 (3.9)	
Other	28 (11.1)	107 (7.4)	135 (8.0)		34 (7.0)	70 (14.8)	104 (10.8)	
**Wealth Index**								
Poorest	36 (14.2)	342 (23.8)	378 (22.4)	0.000	130 (26.7)	121 (25.5)	251 (26.1)	0.053
Poorer	44 (17.4)	324 (22.6)	368 (21.8)		107 (22.0)	90 (19.0)	197 (20.5)	
Middle	51 (20.2)	262 (18.2)	313 (18.5)		105 (21.6)	108 (22.8)	213 (22.2)	
Richer	47 (18.6)	274 (19.1)	321 (19.0)		72 (14.8)	101 (21.3)	173 (18.0)	
Richest	75 (29.6)	235 (16.3)	310 (18.3)		73 (15.0)	54 (11.4)	127 (13.2)	
**Subnational Levels (B/N)**								
Barisal/Province 1	38 (15.0)	156 (10.9)	194 (11.5)	0.009	63 (12.9)	65 (13.7)	128 (13.3)	0.000
Chittagong/Madhesh	46 (18.2)	251 (17.5)	297 (17.6)		56 (11.5)	163 (34.3)	219 (22.8)	
Dhaka/Bagmati	38 (15.0)	203 (14.1)	241 (14.3)		61 (12.5)	33 (7.0)	94 (9.8)	
Khulna/Gandaki	38 (15.0)	135 (9.4)	173 (10.2)		58 (11.9)	33 (7.0)	91 (9.5)	
Mymensingh/Lumbini	19 (7.5)	194 (13.5)	213 (12.6)		76 (15.6)	86 (18.1)	162 (16.9)	
Rajshahi/Karnali	18 (7.1)	136 (9.5)	154 (9.1)		72 (14.8)	66 (13.9)	138 (14.4)	
Rangpur/Sudur	27 (10.7)	158 (11.0)	185 (10.9)		101 (20.7)	28 (5.9)	129 (13.4)	
Sylhet	29 (11.5)	204 (14.2)	233 (`3.8)					
**Number of ANC Visits**								
< 4	117 (46.3)	833 (58.0)	950 (56.2)	0.001	80 (16.4)	206 (43.5)	286 (29.8)	0.000
4+	136 (53.7)	604 (42.0)	740 (43.8)		407 (83.6)	268 (56.5)	675 (70.2)	
**PNC within the First Week**								
No	163 (64.7)	580 (40.4)	743 (44.0)	0.000	388 (79.7)	407 (86.1)	795 (82.8)	0.009
Yes	89 (35.3)	857 (59.6)	946 (56.0)		99 (20.3)	66 (13.9)	165 (17.2)	
**Health Facility Delivery**								
Non-Health Facility	65 (26.0)	846 (58.9)	911 (53.9)	0.000	85 (18.1)	249 (56.5)	334 (36.7)	0.000
Health Facility	188 (74.3)	591 (41.1)	779 (46.1)		384 (81.9)	192 (43.5)	576 (63.3)	
**C-Section Delivery**								
No	109 (43.3)	1063 (74.0)	1172 (69.4)	0.000	436 (89.5)	449 (94.7)	885 (92.1)	0.003
Yes	143 (56.7)	374 (26.0)	517 (30.6)		51 (10.5)	25 (5.3)	76 (7.9)	
**Other Substances Applied**								
No	145 (57.3)	963 (67.0)	1108 (65.6)	0.003	433 (88.9)	294 (62.0)	727 (75.7)	0.000
Yes	108 (42.7)	474 (33.0)	582 (34.4)		54 (11.1)	180 (38.0)	234 (24.3)	
**Other Non-Harmful Substances Applied**								
No	166 (65.6)	1081 (75.2)	1247 (73.8)	0.001	487 (100)	468 (98.7)	955 (99.4)	0.013
Yes	87 (34.4)	356 (24.8)	443 (26.2)		0.00	6 (1.3)	6 (0.6)	
**Harmful Substances Applied**								
No	230 (90.9)	1174 (81.7)	1404 (83.1)	0.000	437 (89.7)	307 (64.8)	744 (77.4)	0.000
Yes	23 (9.1)	263 (18.3)	286 (16.9)		50 (10.3)	167 (35.2)	217 (22.6)	

Notes: col = column. P-value calculated using Chi-square test. Due to rounding some column percentages may be slightly off 100%

There were several significant findings when comparing groups that did and did not receive chlorhexidine cleansing in both countries. In Bangladesh 78% of newborns receiving chlorhexidine had a mother with secondary and higher education compared to 62% for newborns not receiving chlorhexidine. In Nepal the percentages were 63% and 44% for newborns receiving and not receiving, chlorhexidine, respectively. There were also significant differences by wealth and subnational areas in both countries. In both countries, more newborns receiving chlorhexidine were in the highest wealth quintile compared to the newborns not receiving chlorohexidine, though this finding was only marginally significant for Nepal. In both countries more newborns receiving chlorhexidine had at least four ANC visits (Bangladesh = 54% vs. 42%; Nepal = 84% vs. 57%) and skilled delivery (Bangladesh = 74% vs. 41%; Nepal = 82% vs. 44%) compared to newborns not receiving chlorhexidine. In both countries a higher proportion of newborns receiving chlorhexidine were born by Cesarean delivery in contrast to newborns not receiving chlorhexidine. There were also significant, but contrasting, findings for the application of other substances. In Bangladesh newborns receiving chlorhexidine were more likely to have another substance applied, and another non-harmful substance applied, but less likely to have a harmful substance applied. In Nepal newborns receiving chlorhexidine were less likely to have any other substance applied than newborns not receiving chlorhexidine.

The results for the multivariable analyses are presented in Table 2. In both countries there was a strong association between health facility delivery and the newborn’s receipt of chlorhexidine after controlling for other factors. In Bangladesh, the OR was 2.39 (95% CI = 1.38–4.12; *p* = 0.002), and in Nepal, the OR was 4.79 (95% CI = 2.98–7.68; *p* = 0.000). In Nepal, there were also significant associations for receipt of chlorhexidine and having at least 4 ANC visits (OR = 2.39, 95% CI = 1.31–3.77; *p* = 0.000) after controlling for other factors. In Bangladesh, newborns born by Cesarean delivery having had a 64% increase in the odds of receiving chlorohexidine compared to those not receiving chlorhexidine (OR = 1.64, 95% CI = 1.05–2.5 *p* = 0.029). In addition, in Bangladesh newborns who had another non-harmful substance applied to the cord, had a 49% increased odds of receiving chlorhexidine (OR = 1.49; 95% CI = 1.05–2.10; *p* = 0.025) compared to newborns not receiving another non-harmful substance. Having a harmful substance applied to the umbilical cord was significantly and negatively associated with chlorhexidine receipt for newborns in Nepal (OR = 0.46; 95% CI = 0.29–0.74; *p* = 0.001). Other key findings included significant differences at the subnational level. Finally, in Nepal newborns from the wealthiest two quintiles were less likely to receive chlorhexidine than newborns in the poorest quintile ((Richest quintile: OR = 0.46; 95% CI = 0.24–0.88; O = 0.020 and richer quintile: OR = 0.38; 95% CI = 0.21–0.69; *p* = 0.001).

**Table 2:** Multivariable Regression Results for Bangladesh and Nepal

Factors	Multivariable Regression Results					
	Bangladesh			Nepal		
	OR	95% CI	*p*	OR	95% CI	*p*
**Maternal Age at Birth (in years)**						
11–19	REF			REF		
20–34	0.78	(0.53, 1.15)	0.209	1.75	(1.09, 2.83)	0.022
35+	0.76	(035, 1.65)	0.489	1.36	(0.31, 5.99	0.604
**Parity**						
1	REF			REF		
2–3	1.38	(0.96, 1.99)	0.082	0.74	(0.50, 1.09)	0.127
4+	1.14	(0.51, 2.52)	0.75	0.46	(0.25, 0.88)	0.019
**Gender of the Child**						
Male	REF					
Female	0.90	(0.67, 1.25)	0.52	1.06	(0.74, 1.51)	0.750
**Mother’s Highest Level of Education**						
None	REF					
Primary	0.83	(0.34, 2.01)	0.679	1.06	(0.60, 1.87)	0.851
Secondary and Higher	1.16	(0.51, 2.64)	0.716	0.99	(0.58, 1.68)	0.971
**Residence**						
Urban	REF					
Rural	1.08	(0.70, 1.66)	0.729	0.71	(0.49, 1.04)	0.081
**Religion (Bangladesh/Nepal)**						
Hinduism						
Islam/Buddhism	REF			0.86	(0.30, 2.46)	0.782
Other	1.24	(0.73, 2.12)	0.429	0.71	(0.36, 1.39)	0.319
**Wealth Index**						
Poorest	REF					
Poorer	1.11	(0.68, 1.83)	0.678	0.84	(0.50, 1.39)	0.497
Middle	1.23	(0.72, 2.10)	0.455	1.02	(0.58, 1.82)	0.937
Richer	1.08	(0.61, 1.93)	0.783	0.38	(0.21, 0.69)	0.001
Richest	1.35	(0.74, 2.48)	0.327	0.46	(0.24, 0.88)	0.020
**Subnational Levels (Bangladesh/Nepal)**						
Barisal/Province 1	REF					
Chittagong/Madhesh	0.55	(0.31, 1.00)	0.050	0.78	(0.41, 1.48)	0.445
Dhaka/Bagmati	0.50	(0.26, 0.96)	0.036	1.94	(1.04, 3.61)	0.037
Khulna/Gandaki	0.70	(0.35, 1.40)	0.311	2.04	(0.96, 4.36)	0.065
Mymensing/Lumbini	0.37	(0.18, 0.75)	0.006	1.07	(0.57, 2.02)	0.833
Rajshahi/Karnali	0.37	(0.16, 0.82)	0.015	1.93	(0.98, 3.82)	0.058
Rangpur/Sudur	0.55	(0.26, 1.16)	0.117	3.04	(1.57, 5.89)	0.001
Sylhet	0.51	(0.28, 0.95)	0.033			
**Number of ANC Visits**						
< 4	REF					
4+	0.86	(0.60, 1.22)	0.399	2.39	(1.51, 3.77)	0.000
**Health Facility Delivery**						
Non-Health Facility	REF					
Health Facility	2.39	(1.38. 4.12)	0.002	4.79	(2.98, 7.68)	0.000
**C-Section Delivery**						
No	REF					
Yes	1.64	(1.05, 2.55)	0.029	1.19	(0.63, 2.26)	0.590
**Other Non-Harmful Substances Applied**						
No	REF			NA		
Yes	1.49	(1.05, 2.10)	0.025			
**Harmful Substances Applied**						
No				REF		
Yes	0.96	(0.56, 1.66)	0.889	0.46	(0.29, 0.74)	0.001

Notes: OR = odds ratio; CI = confidence interval; *p* = *p*-value; REF = reference group; ANC = antenatal care

## Discussion

Neonatal mortality is a tragic event, especially those deaths that could be averted with known interventions. It is imperative that effective interventions are implemented and scaled-up, particularly in low resource environments, where most newborn deaths occur. Chlorhexidine cleansing for umbilical cord care is an intervention that has been shownl to reduce neonatal deaths by preventing omphalitis. Estimated costs for chlorhexidine for newborn umbilical cord cleansing are less than a dollar per dose [26, 27]. Additionally, chlorhexidine has a long-shelf life and does not need to be kept at cold temperatures [27], which helps make the intervention feasible in settings which may lack electricity. While clinical trials have explored the effectiveness of chlorhexidine [6–8, 12, 13], we explored coverage of the intervention in two countries, Bangladesh and Nepal with national policies and population-level data.

Overall coverage of chlorhexidine was 50.7% in Nepal, which implemented a national policy regarding use of chlorhexidine for all live births in 2011 and a scale-up phase beginning in 2014. The government promoted scale-up of the policy within existing programs such as the Community-based Newborn Care Program (CB-NCP) [28]. This program included training of both health workers and Female Community Health Volunteers (FCHV) [18, 29]. Coverage of chlorhexidine was lower in Bangladesh (15%), where the national policy began later in 2014, than in Nepal. Even with national policies in place, scale-up takes time and challenges can arise. Bangladesh initially promoted chlorhexidine use in all settings, but later changed the early focus to deliveries in health facilities. Initial reports indicated that there were some instances where chlorhexidine was being mistaken for eye drops [19]. In order to prevent such misunderstanding, chlorhexidine was later produced in Bangladesh and packaged in a bottle with a purple top to distinguish it from eye and nasal drops [19]. In addition training of Maternal and Newborn Health (MNH) workers in public facilities in Bangladesh took place from June 2015 to September 2016.In some cases, chlorhexidine was not actually available in the health facilities until months after the training [20]. Thus, given that data for this study came from births in the most recent year from the 2017–2018 Bangladesh DHS, it is not surprising that coverage was fairly low at that point in time. A household survey conducted during November 2017 in four districts of Bangladesh found chlorhexidine coverage to be 33% [20], so it is plausible that newer data national household data may also reveal higher coverage.

In both countries the use of a harmful substance for cord cleansing is fairly common at 16.9% for Bangladesh and 22.6% for Nepal: 22.6% According to the latest WHO guidelines [11], this use of harmful substances on the umbilical cord indicates that the promotion of chlorhexidine for umbilical cord cleansing is warranted. While dry cord care is an effective practice, the application of chlorhexidine can be a protective alternative in communities which tend to place a harmful substance on the cord after birth. Chlorhexidine is often available in liquid or gel form, and a qualitative study in Nepal found that caregivers may prefer the gel which can be applied by finger [27]. Caregivers may feel that the action of applying chlorhexidine by finger replicated the action of applying a traditional substance to the umbilical cord [14, 30].

According to the results of our multivariable analyses, there was a strong association between health facility delivery and a newborn’s receipt of chlorhexidine in both countries. Perhaps more attention needs to be paid to ensuring chlorhexidine is available for newborns who are born in non-facility environments. Engaging community health workers and traditional birth attendants (TBAs) could be Having four or more ANC visits was a significant factor for a newborn’s receipt of chlorhexidine in Nepal. In 2012, Nepal began distributing chlorhexidine during ANC visits at health facilities, so that mothers who are unable to make it to a health facility for delivery still have access to chlorhexidine [18]. In Bangladesh, mothers who had a Cesarean section were significantly more likely to have a newborn who received chlorhexidine. This could be due to the fact that these deliveries would be occurring in health facilities, where chlorhexidine is more easily accessible. In Bangladesh having another non-harmful substance applied was significantly associated with receipt of chlorhexidine, while in Nepal having a harmful substance applied was significantly and negatively associated with receipt of chlorhexidine. Findings from our descriptive analyses also indicated that some newborns (9% in Bangladesh and 10% in Nepal) were getting both chlorhexidine and a harmful substance. Qualitative research could be helpful in uncovering reasons why more than one substance is being applied to the cords of some newborns.

In our multivariable regression results both the significance and non-significance of different socio-demographic factors was revealing. There were some differences by sub-national level, perhaps partly due to some areas promoting the intervention earlier than others. At scale-up, however, subnational differences could be addressed so that newborns in all areas have equal access. Mother’s education, urban/rural status, religion and sex of the child were all insignificant predictors of chlorhexidine use. in the multivariable regression analyses. Wealth was not a significant factor in Bangladesh, and in Nepal newborns from the wealthiest households were significantly less likely to receive chlorhexidine than newborns from the poorest households. Poverty is often a strong determinant of access to health care, but chlorhexidine for newborn care, could be one of the few interventions that are accessible regardless of household wealth, mother’s education, religion, sex and urban versus rural residence. However, it could be that families of newborns in wealthier households follow dry cord care, though both countries are aiming for 95% coverage of chlorhexidine [15, 16].

Both countries have plans to monitor and evaluate coverage, scale-up and quality of the intervention. Bangladesh collects data on chlorhexidine for newborn umbilicial cord care from national-level surveys [14] and routine health information systems at regular intervals [16]. In Nepal a chlorhexidine coverage and compliance study in 2017 has revealed that the country had achieved 59% coverage with low coverage for home births compared to facility births [31]. A validation study of chlorhexidine use in Bangladesh and Nepal indicated that almost all newborns observed received chlorhexidine, and in terms of timing 92% received it within one hour of birth [21].

There are some limitations to our analysis. The main limitation is recall bias, as some women may not have remembered or were not told whether chlorhexidine was applied. A validation study found that during exit interviews women who delivered at the health facility under-reported whether their newborns received chlorhexidine compared to observer assessed reporting [21]. This limitation is somewhat mitigated by the unique packaging of chlorhexidine in Bangladesh and the availability of a gel formulation in Nepal. We also excluded the small percentage of women who responded “Don’t Know” to the questions on whether any substance was applied to their newborn’s umbilical cord and limited the time frame to births in the past year. This study is cross-sectional in nature, and thus we can only study associations and not causality. Another key limitation is that based on the available data, Bangladesh had less time for implementation of the national guidelines than Nepal. We hope to replicate this study with newer data as it becomes available.

Despite the limitations, this paper assessed chlorhexidine coverage in two countries with national, albeit different, policies and national data. We find that there is a strong association with access to the intervention and health facility delivery, so expanding community-based programs may be an effective means to reach more newborns who are born in non-facility environments. More research could go into why some newborns are still receiving a harmful substance and why some newborns receive both chlorhexidine and a harmful substance. The fact that wealth was not a significant factor in Bangladesh and that newborns from the poorest households in Nepal had equal or greater access to chlorhexidine, may indicate that this intervention has the potential to be equitable. In addtion maternal education was not a signifcant factor, suggesting the newborns whose mothers had less education were not disadvantaged in receiving the intervention. Other countries that meet the current WHO standard for provision of chlorhexidine could learn from the promotion of chlorhexidine in Bangladesh and Nepal.

## Conclusion

Nepal and Banglaesh have long-standing policies regarding the promotion of chlorhexidine for newborn umbilical cord care and also population level data to assess coverage of the intervention. Nepal, the first country to promote the scale-up of chlorhexidine had a coverage of 50.7%, while Bangladesh had a coverage of 16.9% The application of a potentially harmful substance was relatively high at 16.9% in Bangladesh and 22.6% in Nepal. More research is needed into why some families are still using potentially harmful substances. As Bangladesh, Nepal and other countries work to scale-up chlorhexidine for newborn umbilical cord care, implementing strategies to reach more newborns born in non-facilities environments would be an important programmatic focus. More population-level data on chlorhexidine for newborn umbilical cord care is needed to enable more countries to monitor and evaluate coverage and scale-up.

## Data Availability

The data sources used in the analysis can be accessed at the web link upon request https://www.dhsprogram.com/data/available-datasets.cfm.

## Abbreviations

ANC: antenatal care
CB-NCP: community based newborn care program
CI: confidence interval
DHS: demographic and health survey
EA: enumeration area
FCHV: female community health volunteer
MNH: maternal and newborn health
OR: odds ratio
WHO: World Health Organization

## References

[1] United Nations Inter-agency Group for Child Mortality Estimation (UNIGME) (2023). Levels & trends in Child Mortality: Report 2022, estimates developed by the United Nations Inter-agency Group for Child Mortality Estimation.

[2] World Health Organization (WHO). ‘Newborn Mortality-Key Facts’. 2023. https://www.who.int/news-room/fact-sheets/detail/levels-and-trends-in-child-mortality-report-2021.

[3] Painter K, Anand S, Philip K. Omphalitis. StatPearls. Treasure Island (FL). Volume 12. StatPearls Publishing; 2022. 30020710

[4] Mir F, Tikmani SS, Shakoor S, Warraich HJ, Sultana S, Ali SA, Zaidi AK (2011). Incidence and etiology of omphalitis in Pakistan: a community-based cohort study. J Infect Dev Ctries.

[5] Yadav P, Yadav SK (2022). Progress in diagnosis and treatment of neonatal Sepsis: a review article. JNMA J Nepal Med Assoc.

[6] Arifeen SE, Mullany LC, Shah R, Mannan I, Rahman SM, Talukder MR, Begum N, Al-Kabir A, Darmstadt GL, Santosham M, Black RE, Baqui AH (2012). The effect of cord cleansing with chlorhexidine on neonatal mortality in rural Bangladesh: a community-based, cluster-randomised trial. Lancet.

[7] Soofi S, Cousens S, Imdad A, Bhutto N, Ali N, Bhutta ZA (2012). Topical application of chlorhexidine to neonatal umbilical cords for prevention of omphalitis and neonatal mortality in a rural district of Pakistan: a community-based, cluster-randomised trial. Lancet.

[8] Mullany LC, Darmstadt GL, Khatry SK, Katz J, LeClerq SC, Shrestha S, Adhikari R, Tielsch JM (2006). Topical applications of chlorhexidine to the umbilical cord for prevention of omphalitis and neonatal mortality in southern Nepal: a community-based, cluster-randomised trial. Lancet.

[9] Imdad A, Mullany LC, Baqui AH, El Arifeen S, Tielsch JM, Khatry SK, Shah R, Cousens S, Black RE, Bhutta ZA (2013). The effect of umbilical cord cleansing with chlorhexidine on omphalitis and neonatal mortality in community settings in developing countries: a meta-analysis. BMC Public Health.

[10] World Health Organization (2014). WHO recommendations on postnatal care of the mother and newborn.

[11] WHO recommendations on maternal and newborn care for a positive postnatal experience. Geneva: World Health Organization; 2022. License: CC BY-NC-SA 3.0 IGO. 35467813

[12] Sazawal S, Dhingra U, Ali SM, Dutta A, Deb S, Ame SM, Mkasha MH, Yadav A, Black RE (2016). Efficacy of chlorhexidine application to umbilical cord on neonatal mortality in Pemba, Tanzania: a community-based randomised controlled trial. Lancet Glob Health.

[13] Semrau KEA, Herlihy J, Grogan C et al. Effectiveness of 4% chlorhexidine umbilical cord care on neonatal mortality in Southern Province, Zambia (ZamCAT): a cluster-randomised controlled trial. Lancet. 2016. 10.1016/S2214-109X(16)30215-7 27693439

[14] National Institute of Population Research and Training (NIPORT), and ICF (2020). Bangladesh Demographic and Health Survey 2017-18. Dhaka, Bangladesh, and Rockville.

[15] New ERA, Ministry of Health, Nepal (2017). Nepal Demographic and Health Survey 2016.

[16] Coffey PS, Brown SC (2017). Umbilical cord-care practices in low- and middle-income countries: a systematic review. BMC Pregnancy Childbirth.

[17] Ministry of Health Nepal (2016). Every newborn action plan.

[18] Government of Bangladesh. Operational plan of maternal neonatal child and adolescent health (MNCAH). Ministry of Health and Family Welfare; 2016.

[19] USAID. Chlorhexidine. Navi (Cord) Care Program. US Agency for International Development. 2015. https://2017-2020.usaid.gov/documents/1861/fact-sheet-chlorhexidine-%E2%80%9Cnavi%E2%80%9D-cord-care-program.

[20] JSI. Scaling-up the Use of Chlorhexidine for umbilical cord care: Nepal’s experience. JSI Research & Training Institute, INC; 2017.

[21] Healthy Newborn Network (HNN). Bangladesh’s experience of chlorhexidine utilized through a dropper bottle. Healthy Newborn Netw. 2019. https://www.healthynewbornnetwork.org/blog/bangladeshs-experience-of-chlorhexidine-utilized-through-a-dropper-bottle/.

[22] Callaghan-Koru JA, Khan M, Islam M, Sowe A, Islam J, Billah SM, Mannan II, George J, Bangladesh Chlorhexidine Scale Up Study Group (2019). Implementation outcomes of the national scale up of chlorhexidine cord cleansing in Bangladesh’s public health system. J Glob Health.

[23] Zaman SB, Siddique AB, Ruysen H, Kc A, Peven K, Ameen S, Thakur N, Rahman QS, Salim N, Gurung R, Tahsina T, Rahman AE, Coffey PS, Rawlins B, Day LT, Lawn JE, Arifeen SE, EN-BIRTH Study Group (2021). Chlorhexidine for facility-based umbilical cord care: EN-BIRTH multi-country validation study. BMC Pregnancy Childbirth.

[24] World Population Review. 2023. https://worldpopulationreview.com/.

[25] World Data Info. 2023. https://www.worlddata.info/.

[26] PATH. (2013). Breakthrough innovations that can save women and children now. from http://www.path.org/innovations2015/.

[27] Health Communication Capacity Collaboration. About Chlorhexidine. Johns Hopkins University. .1%25%20chlorhexidine%20digluconate%2 C%20a%20low,less%20than%20%241%20per%20dose; 2023. https://sbccimplementationkits.org/demandrmnch/about-chx/#:~:text=7.

[28] Pradhan YV, Upreti SR, Pratap KCN (2012). Newborn survival in Nepal: a decade of change and future implications. Health Policy Plan.

[29] KC A, Thapa K, Pradhan YV, KC NP, Upreti SR, Adhikari RK, Khadka N, Acharya B, Dhakwa JR, Aryal DR, Aryal S, Starbuck E, Paudel D, Khanal S, Devkota MD (2011). Developing community-based Intervention Strategies and Package to Save newborns in Nepal. J Nepal Health Res Counc.

[30] Hodgins S, Pradhan Y, Khanal L, Upreti S, Pratap Kc N (2013). Chlorhexidine for umbilical cord care: game-changer for newborn survival?. Glob Health Sci Pract.

[31] Oyloe P, Khanal L, Hodgins S, Pradhan ST, Dawson P (2017). Innovative product Development Partnership reduced neonatal mortality in Nepal through Improved Umbilical Cord Care. Health Aff (Millwood).

